# The Implications of Metabolic and Bariatric Surgery on Psychosocial and Relational Health: A Narrative Review

**DOI:** 10.1007/s11695-025-08193-w

**Published:** 2025-09-06

**Authors:** Tommaso Dionisi, Vittorio De Vita, Giovanna Di Sario, Lorenzo De Mori, Antonio Gasbarrini, Giovanni Gasbarrini, Giovanni Addolorato

**Affiliations:** 1 Department of Medical and Surgical Sciences, Internal Medicine Unit, Columbus-Gemelli Hospital, Fondazione Policlinico Universitario Agostino Gemelli IRCCS, Rome, Italy; 2 Department of Medical and Surgical Sciences, CEMAD - Digestive System Disease Center, Internal Medicine and Gastroenterology Unit, Fondazione Policlinico Universitario Agostino Gemelli IRCCS, Rome, Italy; 3 Department of Woman and Child Health and Public Health, Fondazione Policlinico Universitario A. Gemelli IRCCS, Rome, Italy; 4 Unit of Addiction Treatment, ASL Roma 4, Local Health Authority, Bracciano, Rome, Italy; 5 Department of Translational Medicine and Surgery, Università Cattolica del Sacro Cuore, Rome, Italy

**Keywords:** Obesity, Quality of life, Family support, Community support, MBS, Weight loss, Social networking

## Abstract

Obesity is a globally prevalent condition associated with elevated morbidity and mortality. Metabolic and bariatric surgery offers a definitive treatment for class III (BMI > 40) obesity, achieving substantial, enduring weight loss and improving metabolic health. Despite extensive research on the physical benefits, comparatively fewer reviews investigate the psychosocial and relational changes accompanying these procedures. This narrative review examines how such procedures affect partner relationship quality, sexual function, and broader social integration, aiming to synthesise current findings on key factors like self-esteem, body image, and family support in the recovery process. It further discusses how strong social networks can bolster long-term weight management and psychological outcomes. By viewing these multifaceted changes through a holistic, family-centred lens, the review highlights the interdependence of emotional, familial, and community support systems in optimizing postoperative results and sustaining improvements in quality of life.

## Introduction

### Background and Epidemiology

Obesity is a chronic, multifactorial disease characterised by pathological excess adipose tissue that undermines physiological function and increases morbidity and mortality [1]. Globally, it is the fifth leading cause of death, affecting people of all ages—from adults to teenagers and even children [2]. To identify and categorise obesity, both the World Health Organization (WHO) and the Centers for Disease Control and Prevention (CDC) use the body mass index (BMI) [3].

BMI remains the pragmatic anthropometric index used by both the WHO and CDC. Overweight spans 25.0–29.9 kg m⁻^2^, whereas obesity is subdivided into class I (30.0–34.9 kg m⁻^2^), class II (35.0–39.9 kgm⁻^2^), and class III (≥ 40 kg m⁻^2^).

### Health Burden and Pathophysiology

While traditionally obesity has been attributed primarily to excessive food intake, recent research highlights a broader set of contributing factors [4]. Genetics, environment, and socioeconomic status also play substantial roles in its development, making obesity a complex and multidimensional public health issue. Individuals with higher BMIs are at increased risk for a range of health problems, including cardiovascular diseases and chronic conditions such as type 2 diabetes, high cholesterol, hypertension, coronary heart disease, stroke, and certain cancers [5, 6]. When BMI reaches class III levels, it can adversely affect nearly every organ system, significantly reducing both life expectancy and quality of life [7]. Beyond physical health challenges, obesity is closely linked to psychological issues, such as anxiety, depression, and disordered eating, often exacerbated by societal weight discrimination and negative body image.

Psychiatric comorbidity—the coexistence of a Diagnostic and Statistical Manual of Mental Disorders, 5th ed. (DSM-5) mental disorder such as major depression, generalised anxiety, binge-eating, or substance-use disorder with obesity—is highly prevalent in candidates for metabolic and bariatric surgery (MBS). A large meta-analysis reported that 50–60% of individuals assessed for surgery met criteria for at least one current axis-I condition, including 30–40% with mood disorders, 15–25% with anxiety disorders, and 8–20% with binge-eating pathology [8]. Whether these disorders are causal or merely correlated remains debated: some authors cite shared neuroendocrine pathways and the psychosocial burden of weight stigma, whereas others point to referral bias and socioeconomic adversity [9]. In light of this uncertainty, routine, standardised mental-health screening at baseline and at every postoperative visit—coupled with prompt referral to specialist care—is recommended as part of an integrated biopsychosocial follow-up pathway.

#### Current Treatment Landscape

In recent years, pharmacotherapy options such as glucagon-like peptide-1 (GLP-1) receptor agonists have expanded treatment approaches, yet these medications typically perform best when combined with a healthy diet, regular physical activity, and behavioural changes [10, 11]. Recent evidence confirms that weekly subcutaneous semaglutide 2.4 mg and the investigational oral formulation 50 mg both induce ~ 15% mean weight loss at 68 weeks, while the dual glucagon-like peptide-1/glucose-dependent insulinotropic polypeptide (GLP-1/GIP) agonist tirzepatide achieves 15–21% reductions across the SURMOUNT trials [12–14]. These incretin-based therapies are reshaping non-surgical obesity management and may eventually influence patient selection for surgery. Because the present review concentrates on psychosocial outcomes after MBS, no further pharmacological analysis is provided.

Despite such advancements, MBS remains the primary treatment for individuals with class III obesity. Current American Society for Metabolic and Bariatric Surgery/International Federation for the Surgery of Obesity (ASMBS/IFSO) guidelines [15, 16] recommend MBS for adults with a BMI ≥ 35 kg m⁻^2^ regardless of comorbidities, and for those with BMI 30–34.9 kg m⁻^2^ when metabolic disease is present. In Asian populations, the action point is a BMI ≥ 27.5 kg m⁻^2^. Among the most common surgical options are sleeve gastrectomy (SG) and Roux-en-Y gastric bypass (RYGB) [17]. Recently, newer, less invasive techniques—such as the BioEnterics Intragastric Balloon (BIB) and endoscopic sleeve gastroplasty (ESG)—have been developed. These endoscopic methods offer benefits such as lower procedural risk and reversibility, though they may not be as effective as traditional surgery in achieving sustained weight loss [18].

#### Psychosocial Impact and Aim of the Review

MBS achieves durable weight loss and simultaneously improves key metabolic parameters—blood pressure, glycemia, and lipid profile—thereby lowering cardiovascular risk. The physical and metabolic benefits of MBS are well established; however, considerable psychosocial and relational transformations may also occur. Many patients receive positive feedback on their appearance, a response that often raises self-esteem and body image. For some, however, praise can be unsettling when external perceptions clash with their own self-view [19]. Interpersonal roles and expectations—within friendships, family dynamics, and partner relationships—can change profoundly as patients adapt to new lifestyle habits and self-perceptions [20].

Although popular perceptions still frame obesity as a matter of willpower, converging genetic, endocrine, and neuro-metabolic evidence justifies MBS as a treatment option for adults with treatment-refractory class III obesity [20]. Nevertheless, relatively few reviews address how postoperative changes affect psychosocial domains—particularly partner relationship quality, sexual function, and broader social networks. This narrative review aims to fill that gap by examining the multifaceted psychosocial shifts and highlighting their critical influence on overall patient satisfaction, adherence, and quality of life.

## Methodology

Because psychosocial studies following MBS vary widely in design, measures, and follow-up—rendering a rigorous systematic review or meta-analysis infeasible—we adopted a narrative review approach. Comprehensive searches in PubMed/MEDLINE were conducted on 15 December 2024 and refreshed on 1 July 2025, with retrieval limited to English-language articles published from 1 January 2000 onward—the era that marks both the advent of modern laparoscopic techniques and the first validated psychosocial assessment tools.

For the electronic search, we combined two concept blocks.

The first block captured the surgical exposure and included variations such as “metabolic surgery”, “bariatric surgery”, “weight-loss surgery”, “gastric bypass”, “sleeve gastrectomy”, “one-anastomosis gastric bypass”, “intragastric balloon”, and “endoscopic sleeve gastroplasty”.

The second block addressed psychosocial outcomes and used terms like “psychosocial”, “psychological”, “mental health”, “self-esteem”, “body image”, “quality of life”, “social support”, “family”, “relationships”, “romantic relationship”, “sexual function”, and “social network.”

Databases were queried by pairing terms from the surgical block with every term from the psychosocial block, ensuring that each retrieved record contained at least one word from each group.

Studies were eligible if they enrolled adults (≥ 18 years) who had undergone SG, RYGB, one-anastomosis gastric bypass (OAGB), or an accepted endoscopic alternative such as BIB or ESG. Eligible reports also had to present at least one psychosocial, relational, or sexual-health outcome assessed 12 months or more after surgery. Finally, we limited inclusion to peer-reviewed randomised controlled, cohort, or cross-sectional studies and, where possible, favoured samples of at least 100 participants to maximise statistical precision.

We excluded pediatric populations, case reports or very small series, conference abstracts, dissertations, and other grey literature, as well as studies confined to peri-operative complications without longer-term psychosocial data.

**Fig. 1:**
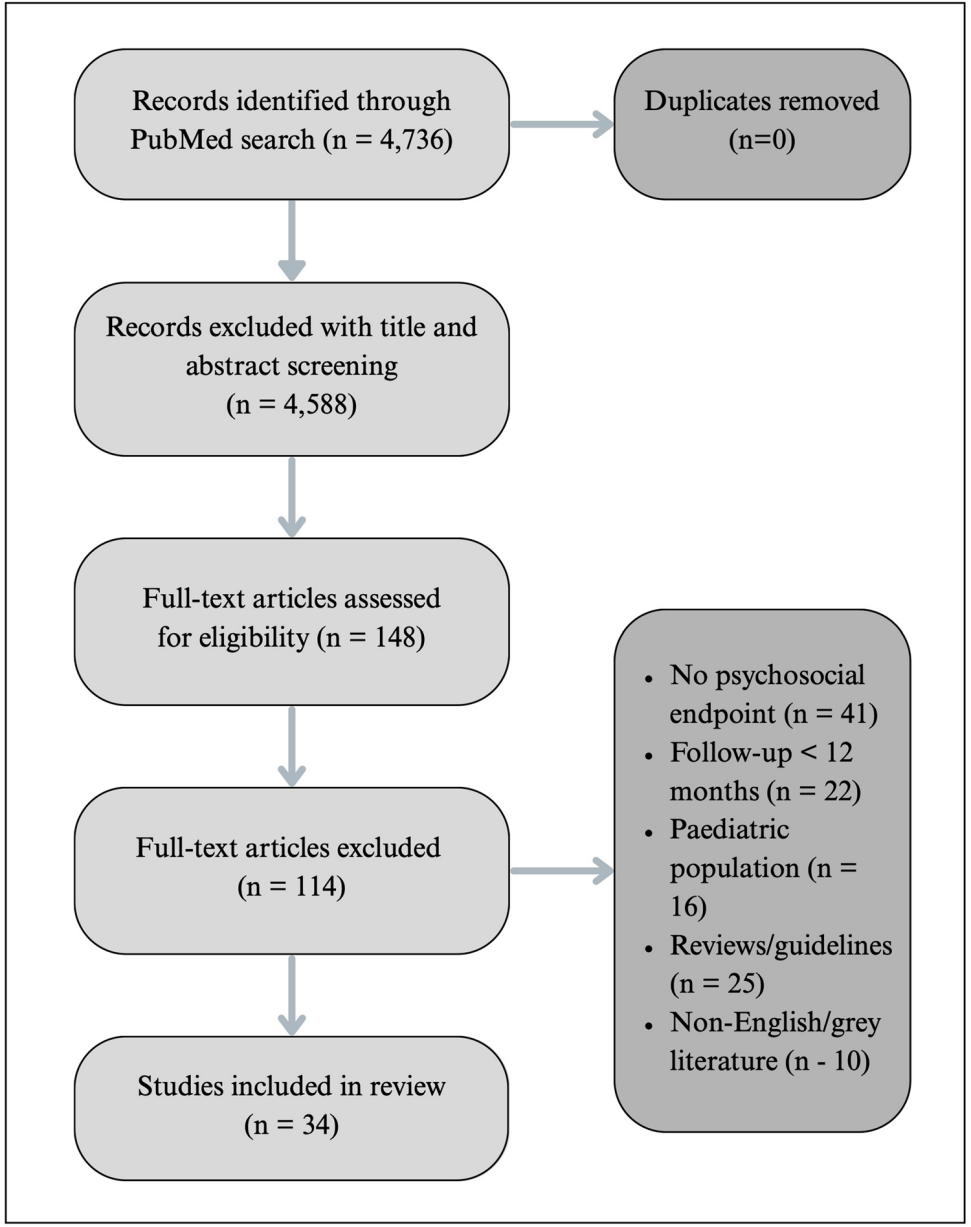
Flow of study selection

Given the marked heterogeneity in study design and endpoints, no formal risk-of-bias tool was applied; instead, higher-level evidence (multicentre cohorts, RCTs, meta-analyses) was qualitatively weighted when formulating conclusions [21]. The study selection process is illustrated in Fig. 1.

## Core Psychosocial Changes After MBS

Following MBS, patients often experience a range of psychosocial changes that can significantly influence their daily lives and relationships (Table 1). For example, weight loss and positive reinforcement from others frequently lead to enhanced self-esteem and body image. Some patients, however, feel uneasy when positive feedback clashes with their self-perception [22]. Another notable shift is a reduction in weight stigma, as visible weight loss tends to lessen external prejudice. Nevertheless, misconceptions persist—chief among them the notion that surgery constitutes an unjustified shortcut [23]. Patients frequently describe heightened social participation, attending events more readily, resuming former hobbies, and benefiting from improved mobility. Finally, shifting roles and expectations may emerge, as newfound energy or personality changes surprise partners, family members, or friends––particularly if previous interactions were shaped around the patient’s obesity [24]. Taken together, these foundational shifts can profoundly reshape intimate relationships, family dynamics, and broader social ties. In many cases, family members or friends may experience jealousy or discomfort, highlighting the importance of open communication, ongoing support, and a holistic approach to postoperative care. In a prospective study of 5749 adults undergoing MBS, self-esteem scores (measured by the Impact of Weight Quality of Life questionnaire) increased from a mean of 33.6 preoperatively to 77.5 at 12 months post-surgery. The increase was consistent across surgery types and demographic groups, with the greatest gains among those with the most weight loss [25]. Such shifts often play a pivotal role in how patients navigate partner relationships, as detailed below.

**Table 1:** Psychosocial domains investigated after metabolic and bariatric surgery

Domain	Most frequent variables/outcomes	Post-MBS trajectory^a^	Key sources
Self-esteem and body image	• Global self-esteem	Rapid improvement within 6–12 months; stabilisation or slight decline after 3–5 years, depending on weight-loss maintenance.	[21–23, 36]
	• Body-image dissatisfaction		
	• Shame/weight concern		
Mental health and HRQoL	• Anxiety, depression, binge-eating	Depressive and anxious symptoms ↓ during the first year; possible re-emergence > 24 months without psychological follow-up; HRQoL ↑ in a sustained manner.	[8, 9, 25, 26]
	• Health-related quality of life (IWQOL-Lite, SF-36)		
	• Use of mental-health services		
Stigma and social participation	• Weight-bias internalisation (WBIS)	Perceived stigma ↓ and social participation ↑; benefit modulated by peer and family support.	[19, 21, 39, 63]
	• Social avoidance/engagement		
	• Satisfaction with social roles		
Couple and family relationships	• Couple quality/stability	Biphasic trend: ~ 2/3 of partners report improved intimacy and support; increased separations/divorces mid-term.	[32–34, 37, 38, 55, 60]
	• Conflict/support		
	• Family adaptation		
Sexuality and reproductive function	• Female (FSFI) and male (IIEF) sexual function	Marked recovery of sexual function within 12 months; hormonal normalisation in men; benefits sustained to 4 years if weight remains stable.	[41, 44, 45, 47, 48]
	• Sex hormones/fertility		
	• Partner sexual satisfaction		
Work role/productivity	• Return-to-work rate	Higher employment rate and productivity within 2 years post-surgery, especially in patients with strong family support.	[30, 54, 56]
	• Presenteeism/absenteeism (WPAI)		
	• Work self-efficacy		

^a^General trend derived from the narrative synthesis of the evidence

After MBS, most patients report marked improvements in health-related quality of life (HRQoL) and mental well-being; however, the three most common procedures follow slightly different psychosocial trajectories. SG is often perceived as the least anatomically extensive option and delivers rapid early gains. During the first postoperative year, generic HRQoL instruments such as the 36-Item Short Form Health Survey (SF-36) and EuroQol 5-Dimension (EQ-5D) show the steepest rises in the domains “physical functioning” and “bodily pain”. Self-esteem rises in parallel with weight loss [26]. At 5 years, the multicentre sleeve bypass randomised trial found overall HRQoL indistinguishable from RYGB, yet sleeve patients scored slightly worse on reflux-specific questionnaires, and those who experienced partial weight regain described a dip in body-image confidence [27].

RYGB tends to produce deeper and more durable weight reduction than SG and is less prone to gastro-oesophageal reflux; these features translate into marginally higher satisfaction in the physical-health domains of HRQoL [28]. Psychological benefits—declines in depression and anxiety, rises in vitality—are numerically similar to SG, but many patients interpret the additional weight loss as evidence of personal efficacy, which, in turn, reinforces social engagement and relationship satisfaction [28].

Evidence for OAGB has expanded markedly over the past two years. In a Brazilian cohort, 94% of patients judged their two-year result “good” or “excellent” on the Bariatric Analysis and Reporting Outcome System (BAROS)—a composite that integrates excess-weight loss, comorbidity remission, quality of life, and complications—with the largest gains seen in self-esteem and work capacity [29]. A single-blinded randomised trial that extended observation to five years reported HRQoL on a par with both SG and RYGB; OAGB produced greater weight loss and less acid reflux than SG, although bile reflux emerged as a new source of gastrointestinal discomfort and occasional worry [30].

Taken together, all three operations attenuate obesity-linked psychological distress by lowering body weight, comorbidity burden, and social stigma. RYGB and OAGB, by virtue of their stronger and more durable weight curves, often confer the greatest long-term gains in self-image, whereas SG offers comparable mental-health relief with fewer anatomical alterations but remains more vulnerable to late reflux and weight recidivism. These nuances reinforce the importance of matching procedure choice to each patient’s clinical profile, lifestyle constraints, and psychosocial priorities, and of providing sustained behavioural follow-up to preserve the early mental-health benefits.

## Obesity and Romantic Relationships

Building on these underlying psychological shifts, MBS can reshape romantic relationships in both positive and challenging ways. Studies indicate that individuals who were single before surgery are more likely to enter new relationships postoperatively, while established couples may see either strengthened bonds or a heightened risk of separation or divorce. Factors such as improved self-esteem, altered physical appearance, and changed social activities contribute to these dynamics. Open communication between patients and partners is crucial for managing expectations around weight loss, body image, and possible role adjustments [31]. Counselling can help address interpersonal challenges, particularly for couples negotiating evolving sexual attraction, lifestyle changes, or emotional pressures [32]. Partners with minimal exposure to the patient’s postoperative experience may harbour misconceptions or unrealistic expectations. Encouraging them to participate in pre- and postoperative education fosters greater preparedness and empathy.

Evidence suggests that couples with robust emotional foundations frequently experience improved relationship quality, leveraging the patient’s increased energy for shared health goals or recreational activities. Conversely, couples with longstanding tensions may see these magnified by new demands and stressors. For instance, a study of 54 jejunoileal bypass patients found that 52% reported enhanced satisfaction one-year post-surgery, whereas 6% reported worsened dynamics [33]. Another study highlights that openly communicating about health and dietary shifts correlates with more favourable relational outcomes [34]. Some research points to slightly higher divorce rates among MBS patients within five years, potentially reflecting preexisting relationship strain. Meanwhile, many couples remain stable, and single individuals often report greater success in dating or marriage, possibly linked to greater self-efficacy and increased social engagement [33–35].

In the Swedish Obese Subjects cohort of 2,871 married participants, the cumulative incidence of divorce or separation after MBS was 9.4% at 4 years and 17.1% at 10 years, compared with 5.5% and 11.6% in matched controls; the adjusted hazard ratio (aHR) was 1.28 (95% CI 1.03–1.60, p 0.03). In the same study’s registry arm (Swedish Obesity Surgery Registry), which followed 29,234 gastric-bypass patients, the aHR for divorce versus population controls was 1.41 (95% CI 1.33–1.49, *p* < 0.001) [35].

Younger patients, those with significant weight loss, or previously divorced individuals may be especially prone to new romantic opportunities [36–38]. However, psychiatric medication use or substance addiction can reduce the likelihood of marriage, illustrating how psychological and physical factors intertwine [33]. Cultural or demographic factors may also play a role. Preliminary evidence suggests that socioeconomic status, age, and educational level influence the degree of postoperative relational changes, yet published research remains limited [35]. In some cultures, extended-family involvement or different social norms may further shape how weight loss affects marriage prospects or relationship stability, warranting additional exploration. A US study found a five-year cumulative incidence of divorce among married surgical patients (hundreds of participants) of 8%, compared to 3.5% in the general US adult population, suggesting a higher risk post-surgery [39]. Moreover, heightened self-esteem in one partner can sometimes lead to jealousy or power shifts within the relationship, underscoring the need for ongoing dialogue and, if necessary, professional intervention to address emerging conflicts or vulnerabilities. Common triggers include one partner feeling excluded from new social circles or distressed by changes in shared eating habits [40].

## MBS and Sexual Function

Obesity can negatively influence sexual function through diminished energy, body dissatisfaction, and medical comorbidities such as hypertension or diabetes. Psychological or relational factors like depression and conflict further affect sexual well-being.

MBS can also profoundly affect patients’ sexual health. To date, no review published after January 2022 has collated sexual-function data beyond 24 months for SG, RYGB, and OAGB, leaving long-term outcomes largely unmapped.

Concerns have been raised about intimacy and relationship stability after major weight changes [41].

Weight loss, achieved surgically or via lifestyle interventions, typically yields notable benefits; however, these gains can wane if patients regain significant weight [42]. Postoperatively, individuals frequently report improvements in stamina and physical ease during intimacy, though personal and relational contexts can amplify or dampen these changes [33].

However, studies in female patients suggest that, while overall sexual dysfunction rates may not drastically differ between surgical and non-surgical cohorts, many note enhancements in desire, arousal, and comfort—often tied to improved body image and self-esteem [43]. In women, studies report raised levels of estradiol, testosterone, follicle-stimulating hormone (FSH), luteinising hormone (LH), and sex-hormone-binding globulin (SHBG), correlating with enhanced sexual arousal and satisfaction [44].

Regarding men, recent evidence shows a broadly comparable pattern of improvement, albeit with distinct hormonal and functional nuances. Weight reduction can elevate testosterone and SHGB, modestly improving erectile function and libido [45]. Meta-analytic data confirm that men with class III obesity can see gains in erection quality, sexual desire, and overall satisfaction after MBS, likely owing to hormonal recalibrations [46, 47].

Growing evidence indicates that MBS confer marked sexual-health benefits to men as well as women. The first large synthesis to focus on male patients [48] showed an average + 8-point gain in the total International Index of Erectile Function (IIEF), with meaningful improvements in erectile function, desire, intercourse satisfaction, and overall satisfaction. A more selective meta-analysis of high-quality trials has since confirmed a 5–6-point rise in the International Index of Erectile Function-5 (IIEF-5) and a 39% absolute reduction in erectile dysfunction 12 months after SG or RYGB [49].

Functional recovery is mirrored by rapid endocrine re-calibration: within the first postoperative year, total testosterone typically rises by 5–8 nmol L⁻^1^, SHGB nearly doubles, and prolactin and estradiol fall [50, 51]. As a result, obesity-related hypogonadism resolves in up to 60% of men. Fertility metrics follow a similar trajectory. In the prospective Bariatric Surgery and Sperm Quality (BARIASPERM) study, total sperm count increased by roughly one-quarter, accompanied by better morphology 18 months after surgery, although the effect on motility remained inconsistent [52].

Psychosocially, men report heightened libido, greater body-image confidence, and reduced performance anxiety; however, follow-up beyond three–four years indicates that these gains taper if substantial weight regain occurs, underscoring the need for sustained lifestyle and psychosocial support [48].

Additionally, partners themselves may experience indirect benefits, including diminished caregiving burdens or improved relational satisfaction [53].

While many see improved quality of life, some patients struggle with residual or new psychological distress—such as “addiction transfer,” where food addiction may shift to another behaviour (e.g., alcohol). Others can experience body dysmorphia despite weight loss. These areas warrant ongoing attention and research, particularly when they affect intimate partnerships. Tailored counselling for men and women, respectively, could include screening for hormonal imbalances or addressing fertility concerns.

## MBS, Family and Social Relationships

Family involvement often proves critical for sustained postoperative success. Research points to stronger metabolic outcomes when household members adopt parallel health behaviours, reinforcing the patient’s dietary and activity goals [54, 55]. Active participation by spouses, siblings, or parents in meal preparation, joint exercise, and follow-up visits can markedly strengthen postoperative adherence. Encouraging family participation in presurgical counselling and early postoperative education helps avoid conflicts rooted in misunderstandings [55, 56]. Role realignments might be required if the patient previously handled most cooking but now relies on specific meal plans. Adolescent patients are particularly affected by parenting stress or family dysfunction, as shown by Zeller et al., who found that caregivers’ well-being strongly influences surgical outcomes [57]. As patients gain self-efficacy, some family members may interpret newfound autonomy as selfish, risking tension or estranged relationships [55, 58]. Professional counselling––including monthly family therapy sessions or group workshops––could offer practical coping strategies to preserve positive bonds and ensure a supportive home environment. Key topics might include meal planning, role reallocation, and strategies for emotional support.

Evidence suggests that structured family therapy can reinforce postoperative coping. The bariatric family therapy (BFT) model—six systemic, solution-focused sessions delivered over three months—yielded an additional 5.3% excess-weight loss, a 6-point improvement in family cohesion (family assessment device), and a 9-point rise in Impact of Weight on Quality of Life-Lite (IWQOL-Lite) self-esteem at 12 months versus usual care [59]. These findings indicate that even low-intensity, family-centred coaching can strengthen lifestyle change, psychosocial adjustment, and long-term weight control [59].

Culturally, extended families might share communal meals or caregiving duties, further influencing the patient’s experience. Tailoring family-based interventions to these diverse contexts can bolster success. Despite the well-documented physical benefits, MBS often entails extensive psychosocial adjustments. Healthcare providers should adopt an integrated approach that addresses physical, emotional, and social factors [60, 61]. A robust social support network—encompassing family, friends, community organizations, and online forums—strongly correlates with improved weight maintenance and psychological well-being. Conversely, social or household dysfunction can undermine progress [62]. Qualitative interviews with bariatric patients in the Michigan MBS Collaborative revealed key themes: changing self-perception, how others perceive them, and evolving relationships [40, 63]. Such insights underscore the delicate interplay between individual transformation and broader social acceptance. As Thonney et al. [64] note, an integrative framework that unites surgical care with mental health support and consistent follow-up can foster more durable outcomes. Patients who join online support groups or in-person communities may find greater accountability and motivation. Meanwhile, those lacking social reinforcement or facing negative judgments may struggle with maintaining lifestyle changes over time. A short example from qualitative studies might be a patient describing how public compliments triggered feelings of anxiety or pressure to maintain rapid weight loss.

## Conclusions

Obesity is a widespread condition linked to considerable morbidity and mortality. Although newer pharmacological treatments show promise, MBS remains the most definitive intervention for individuals with class III obesity, offering significant metabolic and cardiovascular benefits. Endoscopic procedures exist but tend to produce more modest long-term weight results compared to traditional surgery. Beyond the physical realm, MBS can deeply transform partner relationships, sexual well-being, and family dynamics.

### Psychological Implications

Some patients encounter heightened intimacy and strengthen bonds, while others grapple with jealousy, altered social roles, or increased rates of marital dissolution. Similarly, effective family engagement can enhance lifestyle adherence, yet unresolved familial tensions may escalate during the recovery period.

Additionally, robust social networks––including friends and specialised support groups––provide crucial emotional reinforcement and motivation to maintain healthy behaviours. A holistic model addressing both physical and psychosocial aspects of MBS is crucial. This involves recognizing the interaction between emotional support, familial involvement, cultural factors, and community engagement. This approach promotes sustainable weight loss, psychological well-being, and enriched interpersonal relationships, enabling patients to thrive post-surgery.

### Limitations

This review is narrative in design. Although the search spanned PubMed, it did not follow a PRISMA protocol and may therefore be subject to selection bias. The underlying evidence is heterogeneous: most primary studies are single-centre cohorts that vary in procedure mix, baseline BMI, follow-up duration, and psychosocial instruments. More than two-thirds report outcomes shorter than five years, limiting insight into the durability of change or late adverse dynamics such as relationship dissolution. Fewer than 30% employed both a generic (SF-36, EQ-5D) and a bariatric-specific (BAROS, IWQOL) scale, hampering direct comparison, and no formal risk-of-bias appraisal was undertaken. These caveats mean that our conclusions on quality of life, mental health, and relational outcomes should be considered hypothesis-generating rather than definitive.

Future research should prioritise multicentre prospective designs with at least 10 years of follow-up, consistent use of validated psychosocial batteries, and stratification by sex, age, and procedure to clarify long-term trajectories.

Meanwhile, we endorse post-surgical care pathways that integrate surgeons, dietitians, mental-health specialists, and social workers to monitor and support the psychological as well as metabolic course of MBS patients.

## Data Availability

No datasets were generated or analysed during the current study.
